# Editorial: Breastfeeding's role in shaping infant gut microbiota for long-term health

**DOI:** 10.3389/fmicb.2025.1659036

**Published:** 2025-08-13

**Authors:** Rozeta Sokou, Aikaterini Konstantinidi, Stavroula Parastatidou

**Affiliations:** ^1^Department of Neonatology Aretaieio Hospital, National and Kapodistrian University of Athens, Athens, Greece; ^2^Neonatal Intensive Care Unit, Nikaia General Hospital “Aghios Panteleimon”, Piraeus, Greece; ^3^Neonatal Intensive Care Unit, “Elena Venizelou” Maternity Hospital, Athens, Greece

**Keywords:** infant gut microbiota, microbiome, breastfeeding, neonatal gut colonization, maternal milk

Gut microbiota, often referred to as a “second brain,” is an extremely complex ecosystem of microorganisms, including bacteria, viruses, and fungi, that play an active role in numerous physiological processes. Although the term “second brain” originally described the enteric nervous system, it is now also used for gut microbiota due to its impact on brain function via the gut-brain axis, including neurotransmitter and cytokine production (Mpakosi et al., 2025, 2024; Martin et al., 2018). The term “microbiome” incorporates all the genes and genomes of the microorganisms colonizing a host, as well as their composite interactions with the host and its environment (Sokou et al., 2024; Gilbert et al., 2018; Lynch and Pedersen, 2016). Recently, comprehension of the role of microbiota has considerably increased, particularly regarding the developmental burst of the first year of life, which sees rapid microbial colonization. While the fetal environment was traditionally considered sterile, newer studies indicate that colonization may begin even in utero, as microorganisms are transferred through the placenta (Vatanen et al., 2022; Yassour et al., 2018; Younge et al., 2019; Singh and Mittal, 2020). The perinatal and early post-natal establishment of gut microbiota is a crucial factor for the long-term health of the individual. This process is affected by genetic, environmental, and nutritional factors, and the infant's nutrition is of paramount importance. Compared to formula, breastfeeding significantly contributes to microbial synthesis differentiation, diversity, and metabolic activity. Maternal milk (MM) contains numerous beneficial microorganisms, prebiotics, immunologic factors, and bioactive molecules, which synergistically shape a microbial environment to be protective and supportive for the developing infant's immunologic system.

Functional proteins of MM differ quantitatively among mothers and dynamically change levels during lactation (Liu et al., 2019; Verd et al., 2018). These proteins are biologically important for the early development of the infant, although the way they impact gut microbiota is still not fully explored. To investigate this association, Xi, Liang et al. conducted a prospective trial in mother-infant dyads, analyzing the profiles of nine MM functional proteins and the composition of infantile gut microbiota at 42 days and 3 months after birth. Sequencing of 16S rRNA revealed significant correlations between specific proteins, including osteopontin (OPN) and k-casein, and abundance of bacterial species, such as *Parabacteroides distasonis* and *Clostridium butyricum*. According to the results, early milk proteins had a more intense impact on gut microbiota. Additionally, metabolic paths potentially related to the regulation of enzymic levels and metabolism of amino acids, pyruvate, and lipid acids were detected. These findings emphasize the potential regulatory role of MM proteins in early infantile immunologic maturation and support their probable involvement in designing specialized infantile products, including formula milk.

Fat is the second most abundant MM ingredient and covers ~50% of the infant's energy requirements (George et al., 2021). Apart from middle-chain and long-chain fatty acids, MM also contains traces of free short-chain fatty acids (SCFAs). Several gut microbiota species are known to produce SCFAs (Shi et al., 2023), and it has been suggested that SCFAs have a regulatory effect on gut microbiota (Gotoh and Shibata, 2023). In the trial of Xi, Yan et al., the correlation between SCFAs and MM microbiome and the role of SCFAs in the regulation of infantile gut microbiota were investigated. Study results indicated that MM butyric acid (C4) is significantly correlated with the abundance of *Clostridium leptum*, a bacterium crucial for C4 production. Moreover, it was observed that specific Bifidobacterium species possibly cohabitate with the main species producing breastmilk C4, while low breastmilk C4 was associated with increased Salmonella presence in infants. Furthermore, C4 seems to affect metabolic paths related to lysine and arginine synthesis, manifesting a potential symbiotic breastmilk effect on early immunologic and metabolic infantile development.

MM is rich in specific lipids and beneficial microbes and may help prevent neonatal jaundice by enhancing neonatal gut microbiota, reducing inflammation, and modifying bilirubin enterohepatic circulation. Liu et al. assessed the association of microbiota and MM metabolic profile with the presentation of neonatal jaundice. Although the total microbial diversity was not significantly different, breastmilk of mothers whose neonates did not develop hyperbilirubinemia showed increased levels of beneficial bacteria (*Lactobacillus, Akkermansia, Bifidobacterium*) and 24 lipidic metabolites possibly protective against neonatal jaundice.

Lundgren et al. evaluated the relation between MM microbiota and infantile gut microbiota during the first year of life. Findings suggested that the microbial profile of breastmilk correlates with infantile gut microbiota at 6 weeks of life, particularly for infants delivered by cesarean section. Moreover, specific breastmilk microbial communities were associated with communities of infantile gut microbiota in samples collected both contemporaneously and over time. Results indicated that MM microbiota may influence infantile gut microbiota through complex molecular interactions, such as metabolite signaling. Finally, in addition to time, maternal exposure to antibiotics during pregnancy also seems to affect the stability of breastmilk and infant gut microbiota.

Yelverton et al., in a secondary analysis of the MicrobeMom randomized control trial, investigated the potential correlation between maternal wellbeing during pregnancy and breastfeeding practices and microbial diversity of infantile gut at 1 month of age. Indexes of maternal mental health in early and late pregnancy were not associated with neonatal microbial diversity. On the contrary, positive breastfeeding practices at hospital discharge and the first month were correlated with lower microbial diversity, consistent with previous evidence of optimal gut colonization in breastfeeding infants.

The review of Tang et al. focuses on the role of gut microbiota in the pathogenesis of atopic dermatitis in children, highlighting important differences in gut microbiota structure, with reduced microbial diversity, decreased number of beneficial bacteria, and increased harmful bacteria in children with atopic dermatitis compared to healthy controls. Nutrition, environment, delivery mode, use of antibiotics, and gestational diabetes affect gut microbiota and subsequent sensitivity to atopic dermatitis. The significance of MM and human milk oligosaccharides in shaping a healthy and diverse microbiota is emphasized, as is the need for early nutritional intervention in disease prevention. Future research aimed at individualized interventions on gut microbiota for effective prevention and treatment of atopic dermatitis is recommended.

This Research Topic attempted to clarify the complex interaction between breastfeeding and infant gut microbiota, revealing the importance of MM in forming a healthy microbial profile with long-term benefits. The featured articles reveal crucial mechanisms and interventions for early modification of microbiota, contributing valuable information regarding targeted prevention and infantile health optimization strategies.
